# Nephralgia

**Published:** 1885-06

**Authors:** 


### NEPHRALGIA.

Hoping that these lines may call forth, from those more experienced than the writer, the valuable points of their experience, we desire to give a few therapeutic hints for the selection of the homœopathic remedy in treating cases of renal colic.

It is not at all necessary for this purpose that we should give any pathological or clinical history of this disease. The symptoms are very familiar and hardly mistakable.

Treatment: The allopathic method of narcotics, Chloroform, or hot fomentations, etc., we need not here discuss, as we all *know* a better, *quicker*, and more successful method. *Apropos* of this assertion, the writer may mention a case treated by Lycopodium, where the patient was able to resume his professional duties in three days, whereas a previous attack, treated by Morphia, had confined him for two weeks.

The remedies most frequently called for in renal colic are Apis, Arn., Ars., Bell., Berb., Cann-s., Canth., Diosc., Erigeron, Eup-perf., Equisetum, Kali-c., Lyc., Nux-m., Nux vom., Ocimum, Op., Pareira, Piper meth., Sarsap., Sil., Tabac., Uric-ac., Zinc. This list comprises the remedies most often indicated, although, of course, *any* remedy may be required where the symptoms indicate its similarity.

Arnica: Where this remedy is of service we find piercing pains, as if a knife were plunged into the region of kidneys, accompanied by violent tenesmus of bladder; patient is chilly and inclined to vomit. (Dr. Small.)

Arsenic: Patient passes gravel from time to time, causing a dull pain in region of kidneys and down the ureter, accompanied by gastralgia, tickling in urethra, and difficult micturition. These, with the great restlessness common with Arsenic patients, complete the picture.

Belladonna: The pains of this remedy are spasmodic, *crampy*, and straining along the ureter as far as the bladder; the pains come and go quickly; patient's face is apt to be dark red, flushed; maybe, eyes injected, etc.

Berberis: Sticking, digging, tearing, or pulsative pain in region of one or both kidneys; or a violent, cutting, sticking pain from kidney to bladder and urethra; also *red sediment in urine.*

Cann-sat.: Has drawing pain in region of kidneys, extending into inguinal glands, with nauseous sensation at pit of stomach; burning while urinating, yet worse after.

EQUISETUM: Dull pain in region of right kidney, with urging to urinate; slight pain in right kidney, then in left, extending down left side of sacrum.

OCIMUM: Nephitic colic, right side, with violent vomiting every fifteen minutes; she twists about, screams, and groans; red urine, with brickdust sediment after the attack; or blood passed after attack.

PAREIRA BALS.: Has violent pains in bladder and, at times, in back; left testicle is painfully drawn up; pain in thighs, shooting down into toes and soles of feet; strangury so bad has to get on his knees, resting head on floor; urine strongly ammoniacal. Dr. Lippe gives, as the difference between Berb. and Pareira, that the former has pains in hip, latter in thigh; Berb. lacks the strong ammoniacal smell of the Pareira urine.

NUX VOM.: Renal colic, especially of right side, extending to genitals and right leg; worse lying on that side, better on back; painful, ineffectual urging to urinate; urine passes by drops, with burning and tearing; stitches in back when turning, with dull pain while sitting.

TABACUM: Renal colic; violent pains along the ureters; cold sweat and deathly nausea. E. J. L.

